# Endolaser-Induced Cutaneous Necrosis: A Case Report

**DOI:** 10.7759/cureus.99378

**Published:** 2025-12-16

**Authors:** Macarena Olivares, Victor Mercado

**Affiliations:** 1 Orofacial Aesthetic Medicine, Instituto Chileno de Rejuvenecimiento y Optimización de Medicina Estética, Santiago, CHL; 2 Otolaryngology, Instituto de Neurorrehabilitación y Equilibrio, Viña del Mar, CHL

**Keywords:** depigmentation, endolaser necrosis, exosomes, infrared laser, ozone therapy, photobiomodulation, polynucleotides, regenerative medicine

## Abstract

Endolaser techniques emerged in the early 2000s to deliver controlled subdermal photothermal stimulation for collagen remodeling, skin tightening, and facial rejuvenation. Today, endolaser is a minimally invasive option within the spectrum of energy-based therapies aimed at mitigating the inevitable manifestations of aging. By transmitting calibrated laser energy through fine optical fibers into the subdermal plane, the technique promotes neocollagenesis and selective lipolysis while preserving the epidermal surface. Despite a generally favorable safety profile, the nature of the emitted energy may rarely precipitate severe adverse events. Among these, thermal necrosis is particularly distressing due to the depth and extent of dermal injury, often culminating in fibrosis and dyschromia; standard wound care alone is frequently insufficient to restore structural integrity and chromatic balance.

We report a case of thermal necrosis after aesthetic endolaser that was successfully managed with a multimodal regenerative approach.

## Introduction

Laser technology for treating skin disorders has been described for decades, as well as the complications associated with its use. Constant innovation in light-energy technology and improvements in device design have enabled the development of new equipment to meet the growing demands of patients and professionals for safer and more effective laser treatments. Typical treatment errors and adverse events have also been described, highlighting that dermatologic training, extensive experience in laser therapy, and adherence to quality guidelines are prerequisites for safe and successful treatment [1,2].

Some studies in the literature report case series of patients treated with endolaser, evaluating both clinical outcomes and associated complications [3-5]. However, they do not specifically address the management of these adverse events.

For this reason, we propose that a treatment based on different bio-regenerative products may represent a therapeutic alternative for complications such as thermal lesions resulting from the aesthetic use of endolaser.

Within this context, we present a case of endolaser-induced thermal necrosis successfully treated using a multimodal regenerative protocol that targets the four key pillars of regenerative medicine: cellular stimulation, angiogenesis, extracellular matrix restoration, and inflammation modulation [6].

## Case presentation

A 45-year-old female patient presented to our clinic on August 26, 2025, with a thermal injury secondary to an endolaser procedure performed on July 8, 2025. Immediately after the session, the patient noticed a blister along the left mandibular border, which was initially dismissed by the treating practitioner. Over the subsequent days, the lesion progressed into a necrotic eschar. A second healthcare provider later evaluated the injury and recommended surgical grafting; however, the patient declined the invasive approach and sought a regenerative alternative at our clinic.

On examination, the lesion displayed a dry, adherent necrotic crust with mild perifocal erythema and evening-predominant pain rated at 7/10. Pharmacologic analgesia with pregabalin 75 mg daily was initiated, achieving significant pain reduction within one week.

During the first session, 0.5 mL of collagenase was administered for the enzymatic debridement of the necrotic eschar. Adjunctive therapies included ozone therapy at 35-40 µg/mL using an acrylic dome-application system, Renubell® (Yurim Medical Co., Ltd.. Cheongju-si, Chungcheongbuk-do, Republic of Korea), containing 20 mg/mL polydeoxyribonucleotides with microinjections of 0.2 mL with a 30 G needle in the perilesional area and infrared photobiomodulation with 808 nm low-level laser therapy (LLLT) delivering 4 J/cm².

Seven follow-up sessions were conducted, spaced every 10 days, involving a multimodal regenerative approach. Each session included ozone therapy at 45 µg/mL administered through a dome-application system, microinjections of PDRN (0.2 mL per session), infrared photobiomodulation at 808 nm delivering 4 J/cm², and the application of NXO®, a preparation containing 10 billion/mL of Panax ginseng-derived exosomes, delivered via pen microneedling using 0.2 mL per session.

**Figure 1:**
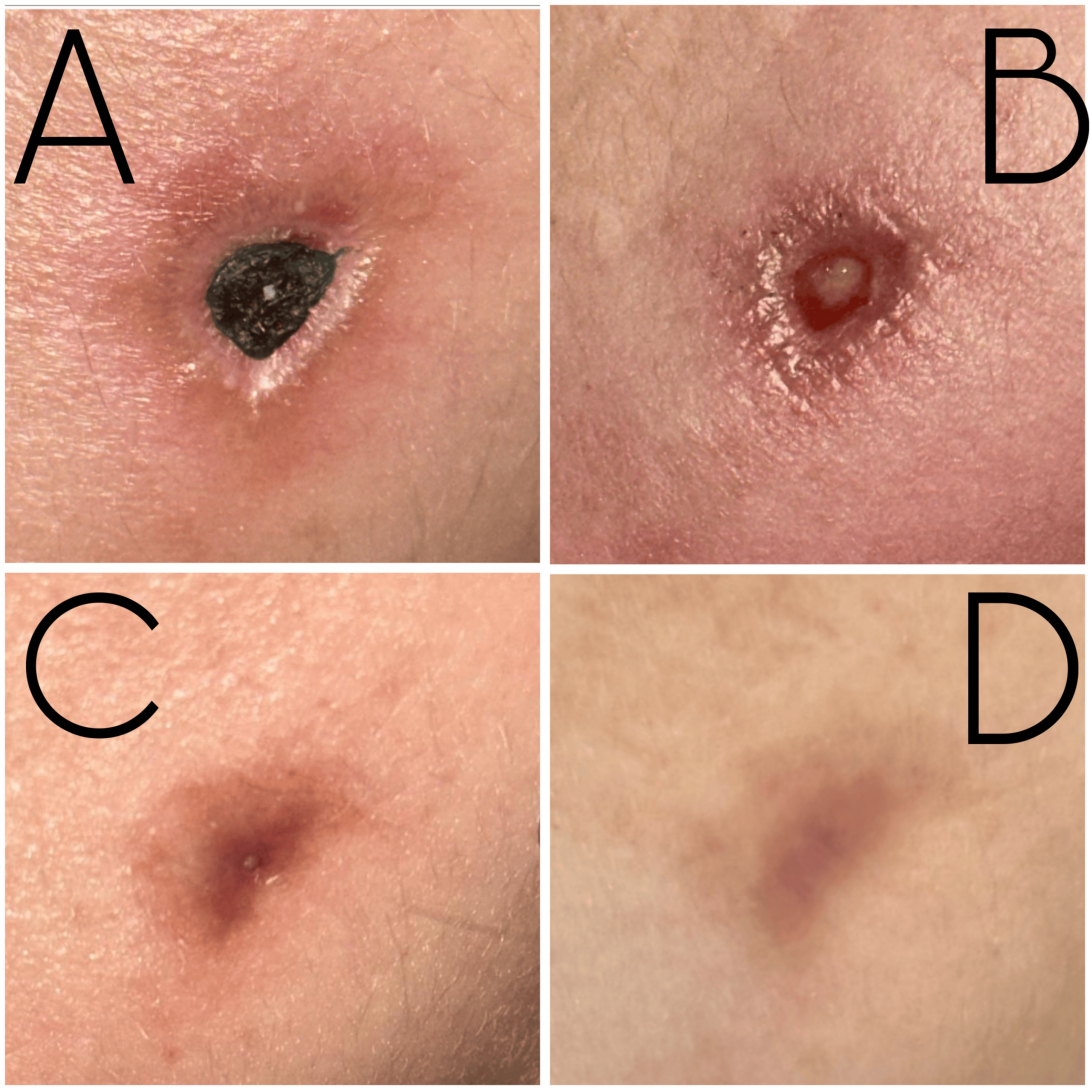
Sequential evolution of thermal burn from endolaser from necrosis to re-epithelialization. (A) Initial presentation showing a well-defined necrotic eschar (1 cm × 5 mm) located on the left mandibular border; (B) exposure of a viable wound bed, (C) progressive marginal closure with homogeneous granulation tissue, and (D) complete epithelial closure with restoration of surface continuity (7 sessions).

The initial necrotic eschar measured 1 cm x 7 mm (Figure 1A). Two days before the debridement, a viable wound bed was observed (Figure 1B). Subsequent sessions showed uniform re-epithelialization and neovascularization, with complete closure by session 5 and no fibrosis (Figure 1C). After session 7, the area exhibited normal elasticity, with full patient satisfaction (Figure 1D). Photography documented the evolution at baseline and after each session. Figure 1 illustrates the progressive recovery of the necrotic burn wound throughout the course of regenerative therapy.

## Discussion

Energy-based devices such as subdermal lasers have gained popularity for their ability to induce dermal tightening, adipose remodeling, and contour enhancement with minimal invasiveness [1,2].

Previous clinical reports also highlight that the safety and efficacy of endolaser techniques depend strongly on operator experience and strict control of energy parameters. Kim DY et al. demonstrated that, in more than 250 cases of lower eyelid laser lipolysis, early minor complications, such as transient sensory disturbances and superficial burns, occurred primarily before energy limits and procedural standards were fully established, after which adverse events declined significantly [3]. Similarly, Senra AB, in a decade-long series of 766 patients treated for localized lipodystrophy, documented a range of complications, including asymmetries, seromas, and occasional infections, some of which required hospitalization [4]. Together, these findings underscore the existence of a procedural learning curve and reinforce the importance of protocol standardization to minimize variability in outcomes and reduce the risk of complications. These reported studies do not analyze in detail the behavior towards thermal burns from endolaser, which is why, in our clinical report, we address this treatment as a real possibility of obtaining good results.

Conventional approaches typically include debridement, topical antimicrobials, corticosteroids, and systemic antibiotics; however, these strategies often fall short in restoring functional or aesthetic integrity, particularly in complex injuries. For this reason, complementary modalities grounded in regenerative medicine are increasingly being explored to accelerate recovery and reduce long-term sequelae [5].

Regenerative medicine is founded on four essential pillars: cellular stimulation, neovascularization, extracellular matrix restoration, and inflammation modulation [6]. These principles guide therapeutic strategies aimed not merely at repairing damaged tissue but re-establishing physiological structure and function through biologically directed healing.

Within this conceptual framework, a multimodal regenerative strategy integrating polydeoxyribonucleotides, exosomes, medical ozone therapy, and infrared photobiomodulation provides synergistic benefits.

Polydeoxyribonucleotide (PDRN)

Through A₂A receptor engagement and cyclic AMP upregulation, PDRN enhances fibroblast proliferation, type I collagen synthesis, and angiogenesis, while modulating inflammation and promoting extracellular matrix (ECM) remodeling [5,7].

Exosomes

Acting as biological messengers, exosomes deliver miRNAs, cytokines, and growth factors that regulate macrophage M2 polarization and re-epithelialization. Wang M et al. highlight their application in wound and burn healing, demonstrating accelerated granulation and reduced scarring [8-11].

Ozone therapy

Ozone induces controlled oxidative preconditioning, enhancing oxygen utilization, stimulating antioxidant enzymes, and increasing growth factor release. Evidence from chronic wound and burn studies shows improved microvascular recruitment and epithelialization [12].

Infrared photobiomodulation (PBM)

Near-infrared wavelengths activate mitochondrial cytochromes, promote nitric oxide release, and increase ATP synthesis, leading to enhanced angiogenesis and cell migration. Randomized controlled trials in diabetic ulcers and second-degree burns report faster healing and improved tissue quality with adjunct PBM therapy [13-15].

Each modality acts on distinct yet interconnected phases of the regenerative cascade, collectively reinforcing the four foundational pillars of tissue repair [6].

## Conclusions

A multimodal regenerative strategy that combines polynucleotides, exosomes, ozone therapy and infrared photobiomodulation, constitutes a therapeutic alternative with good results in the functional and aesthetic recovery of necrotic lesions induced as a complication of the use of endolaser for aesthetic purposes.
